# Prediction for cardiac and pulmonary toxicity in a multicentric cohort of advanced stage NSCLC patients using sub-regions of the heart

**DOI:** 10.1016/j.ctro.2025.100952

**Published:** 2025-04-01

**Authors:** Albrecht Weiß, Steffen Löck, Ting Xu, Zhongxing Liao, Miguel Garrett Fernandes, René Monshouwer, Johan Bussink, Esther G.C. Troost

**Affiliations:** a German Cancer Consortium (DKTK), Partner Site Dresden, and German Cancer Research Center (DKFZ), Heidelberg, Germany; b OncoRay-National Center for Radiation Research in Oncology, Faculty of Medicine and University Hospital Carl Gustav Carus, Technische Universität Dresden, Helmholtz-Zentrum Dresden-Rossendorf, Dresden, Germany; c Department of Radiotherapy and Radiation Oncology, Faculty of Medicine and University Hospital Carl Gustav Carus, Technische Universität Dresden, Dresden, Germany; d Department of Thoracic Radiation Oncology, Division of Radiation Oncology, The University of Texas MD Anderson Cancer Center, Houston, TX, USA; e Department of Radiation Oncology, Radboud Institute for Health Sciences, Radboud University, Medical Center, Nijmegen, the Netherlands; f Department of Medical Physics and Biomedical Engineering, University College London, London, United Kingdom; g National Center for Tumor Diseases (NCT), Partner Site Dresden, Germany: German Cancer Research Center (DKFZ), Heidelberg, Germany; Faculty of Medicine and University Hospital Carl Gustav Carus, Technische Universität Dresden, Dresden, Germany; Helmholtz-Zentrum Dresden-Rossendorf (HZDR), Dresden, Germany; h Helmholtz-Zentrum Dresden-Rossendorf, Institute of Radiooncology-OncoRay, Dresden, Germany

**Keywords:** NTCP, NSCLC, Cardio-oncology, Cardiac adverse events, Pulmonary toxicity

## Abstract

**Purpose:**

Follow-up investigations in locally advanced stage non-small cell lung cancer (NSCLC) patients treated with radiochemotherapy (RCHT) regularly focus around lung toxicity. However, Cardiac Adverse Events (CAE) can occur much earlier in patients than originally anticipated with serious repercussions for patient quality-of-life and survival.

Therefore, here we investigated spatial dependencies of dose within the heart and their correlation with toxicity, with dosimetric parameters of sub-regions of the heart at the focus of this analysis.

Additionally, we aimed to explore the connection between cardiac toxicity and pulmonary toxicity.

**Methods:**

Patient treatment plans with dosimetric data for the lungs and the heart, as well as toxicity data for 502 NSCLC patients treated with either passively scattered proton therapy (PSPT), intensity modulated radiation therapy (IMRT), three-dimensional conformal radiation therapy (3DCRT) or volumetric arc therapy (VMAT) with or without chemotherapy was retrospectively retrieved from prospective clinical studies of three international centers. Cardiac toxicity data was not available for all patients. Data was randomly split into a training set (336) and validation set (166). Statistical analyses were performed using binomial logistic regression.

**Results:**

In univariate modeling, the Mean Lung Dose (MLD) significantly predicted CAE grade ≥ 3 in the training-set (p_MLD_ = 0.02, AUC_train_ = 0.69), which was confirmed in validation (AUC_val,_ = 0.77). No suitable candidates for the construction of multivariate models could be identified. Parameters of the heart and its subregions did not significantly predict CAE grade ≥ 3 in the investigated cohorts. No parameters were found to significantly predict CAE grade ≥ 2 or RP. Finally, no spatial dependency was found in the investigated toxicity data.

**Conclusion:**

The pulmonary dosimetric parameter MLD successfully predicted CAE grade ≥ 3 in a cohort treated with either photons or protons. Cardiac dosimetric parameters as well as spatial parameters did not perform similarly. No parameters were found to significantly predict RP in the investigated cohorts.

## Highlights

• Cardiac toxicity follow-up investigations in NSCLC patients are scarce, although relevant to patient QOL and survival.
• Here, spatial dependencies of dose within the heart and lungs were investigated and prediction models established.
• The MLD was found to predict cardiac adverse events grade ≥ 3 significantly both in training and validation.

## 1 Introduction

Investigations during follow-up visits of locally advanced stage non-small cell lung cancer (NSCLC) patients treated with radiochemotherapy (RCHT) regularly focus on tumor response and toxicities of the lungs [1], [2], [3]. Other organs at risk (OAR), such as the heart, have historically been regarded as radiation-resistant, or toxicity was deemed to occur long after estimated overall survival of these patients. More recent research, however, has shown that cardiac toxicity can occur much earlier than originally anticipated and indeed influences overall survival in those patients [4], [5], [6], [7]. Furthermore, it was shown that dose escalation, although beneficial in terms of improved local tumor control, heavily increases toxicity rates in patients [5], [8], [9]. Consequently, in 2022, a multidisciplinary guideline on cardio-oncology, developed in collaboration with the European Society for RadioTherapy and Oncology (ESTRO) has summarized the evidence to date [10].

Cardiac toxicity typically manifests itself as arrhythmia or pericardial effusion (PE), and in severe cases, even as myocardial infarction or heart failure, posing a threat to patient survival [11]. As treatment strategies and overall survival of NSCLC patients continue to improve, in part due to the introduction of immunotherapy, targeted agents, and new radiation modalities such as proton therapy, considerations regarding cardiac toxicity in these patients have to be reevaluated [12], [13], [14], [15].

Normal-tissue complication probability (NTCP) modeling is used to estimate the incidence of toxicities following radiotherapy. For example, the probability of an individual patient to develop cardiac adverse events (CAE) can be estimated based on comorbidities as well as on dose-volume-histogram (DVH) parameters of the heart [16], [17], [18]. At present, it is unclear, whether these parameters can robustly predict CAE for patients treated with photon as well as with proton beam irradiation. Additionally, increasing evidence has been found of cardiac toxicity contributing to the incidence and severity of pulmonary toxicities, such as radiation pneumonitis (RP), even though the connection and contribution to one another is largely unknown [5], [19], [20], [21].

Thus, in this study, we aimed at identifying parameters predictive of cardiac adverse events in patients treated with either photons or protons based on data from three international centers. In search of spatial dependencies of dose within the heart and their correlation with toxicity, dosimetric parameters of sub-regions of the heart were the core of this analysis. Finally, we investigated parameters for the prediction of the secondary endpoint radiation pneumonitis to gain insight into the connection of cardiac and pulmonary toxicity in NSCLC patients.

## 2 Methods

### 2.1 Patient cohort

Data from three international centers was used for this modelling, i.e., University Hospital Carl Gustav Carus Dresden, Germany (UKD), MD Anderson Cancer Center, Houston, TX (MDACC), and Radboud University Medical Center Nijmegen, the Netherlands (RUMC).

UKD patients had been treated with radiochemotherapy (RCHT) between 2006 and 2021. Patients from MDACC and RUMC had undergone RCHT between 2009 and 2014 and between 2008 and 2014, respectively, in the context of prospective clinical trials [22], [23]. In all centers, the treatment-related toxicities were assessed in weekly follow-up performed by the treating radiation oncologist during RCHT until acute toxicity resolved. For patients from UKD, follow-up was every 2 weeks for the first 10 weeks after completion of RCHT, and every 3 months up to 60 months thereafter. For patients from MDACC, follow-up was at 4–8 weeks after treatment, every 3 months for the first 2 years and every 6 months thereafter, while for patients from RUMC, follow-up visits occurred every 3 months for the first 2 years, then every 6 months for years 3–5. Toxicity was scored using Common Terminology Criteria for Adverse Events (CTCAE) 4.0 for patients from UKD and MDACC, and using the Radiation Therapy Oncology Group (RTOG) Radiation Toxicity Grading for patients from RUMC. To account for systematic errors from using different grading systems, the toxicity data of RUMC patients was converted from RTOG to CTCAE 4.0 [24].

Patients with stage IIA-IIIC NSCLC or with oligometastatic stage IV NSCLC with a solitary brain metastasis (MDACC dataset only) treated with passively-scattered proton therapy (PSPT; MDACC) or with three-dimensional conformal radiation therapy (3DCRT; UKD), intensity-modulated radiation therapy (IMRT; UKD and RUMC) and volumetric-modulated arc therapy (VMAT; RUMC) were included [25]. Staging and treatment planning in all centers was done using 18F-Fluorodeoxyglucose positron emission tomography and computed tomography (FDG-PET-CT).

Anonymized data of patient’s age, gender, treatment schedule and technique, tumor stage, histology, and treatment outcome were retrospectively obtained. To further investigate the relevance of the relative position of the primary tumor in respect to the heart, the tumor location in the thorax was also tested as a predictive parameter. For this, two groups were composed, one with the tumor positions in the right lower lobe (RLL) and left lower lobe (LLL), the other one with the tumor positions in the right middle lobe (RML), right upper lobe (RUL), and left upper lobe (LUL).

Regarding treatment-related toxicity, data on CAE as well as on RP, which had been assessed prospectively was obtained. The cardiac adverse events investigated here include pericardial effusion as well as cardiomyopathy, myocardial infarction, cardiac arrest, and others. Unfortunately, for patients from UKD, CAE data was unavailable, while for patients from MDACC, cardiac comorbidity data was unavailable. Additionally, DVH data of the lungs, of the heart as entire organ and for the cardiac sub-structures was collected (see below).

The Ethics Committee of the Technische Universität Dresden, Germany, approved the retrospective analysis of the obtained data (BO-EK-251062024). Patient data was collected according to the ethics guidelines of the respective centers.

### 2.2 Treatment details

Patients from UKD received 66 Gy in 2 Gy fractions, patients from MDACC received 66 or 74 Gy(relative biological effectiveness [RBE]) in 2 Gy(RBE) fractions, and patients from RUMC received 60 or 66 Gy in 2 Gy fractions. Moreover, all patients received concurrent chemoradiation: patients from UKD received weekly 45 mg/m^2 body surface area (BSA) Paclitaxel and Carboplatin AUC 2, patients from MDACC received weekly 50 mg/m^2 BSA Paclitaxel and Carboplatin AUC 2, and patients from RUMC received 100 mg/m^2 BSA Etoposide and 50 mg/m^2 BSA Cisplatinum weekly.

The gross tumor volume (GTV) consisted of the primary tumor and affected hilar and mediastinal lymph nodes as identified on FDG-PET-CT [26], [27]. The clinical target volume (CTV) enclosed the GTV with an 8 mm margin craniocaudally and 9 mm in lateral as well as anterior-posterior directions (UKD), with a 7 mm circumferential margin (MDACC), or with a 10 mm circumferential margin (RUMC), subsequently corrected for anatomical boundaries. For patients from RUMC and UKD planning target volumes (PTV) were created by an isotropic 5 mm expansion of the CTV. For patients from MDACC range uncertainty was 3.5 %.

Treatment planning was done using the treatment planning system Oncentra OTP v.4.3 (Elekta, Stockholm, Sweden) for patients from UKD, the Eclipse treatment planning system (Varian Medical Systems, Palo Alto, CA) for patients from MDACC, and the Pinnacle planning system (Philips Radiation Oncology Systems, Fitchburg, WI) for patients from RUMC.

### 2.3 Delineations of cardiac subregions

Delineations for sub-regions of the heart (right artery [RA], right ventricle [RV], left artery [LA], left ventricle [LV]) for UKD and RUMC were obtained applying a deep-learning model for automatic contouring [28], which was trained using the atlas by Feng *et al.* [29]. Dosimetric data for patients from UKD and RUMC was obtained from DICOM files using an in-house python script. For patients from MDACC, dosimetric data was provided. For all datasets, maximum and average dose values, and volumes of the organs at risk receiving at least x Gy (VxGy), with x ranging from 0 to 80 Gy in 5 Gy steps, were collected for all organs at risk.

### 2.4 Statistical analyses

The primary endpoint of the investigation was CAE grade ≥ 3 at two years after RCHT. Patients who died or ended follow-up before two years were excluded from the analysis. The analyses of RP grade ≥ 2 and of CAE grade ≥ 2, both two years after treatment, were part of the secondary endpoints of this study. Patients from all datasets were randomly divided into a training- (336 patients) and validation-cohort (166 patients). Statistical analyses were performed using R version 4.2.3 [30]. The data was analyzed using univariable binary logistic regression modeling and two-sample t-testing. Prediction models were established based on our hypotheses for this analysis, and a p-value < 0.05 was considered statistically significant. Furthermore, the area under the receiver operating characteristic curve (AUC) and its 95 % confidence interval were estimated using 10,000 bootstrap samples. For validation of our findings, the AUC and the corresponding confidence interval were estimated in the same way. Validation was deemed successful if the confidence interval did not include the value 0.5.

## 3 Results

The average age of the NSCLC patients was 64, 67, and 63 years for UKD, MDACC, and RUMC, respectively, most patients were male, and the majority (33 %, 35 %, and 42 %, respectively) of tumors was located in the right upper lobe (Table 1). After an average follow-up time of 25.0 (0.2–109) months, 23.4 (0.1–109) months, and 24.0 (0.2–121) months, median overall survival was 19 months, 25 months, and 17 months for patients, respectively.

**Table 1:** Patient, tumor and treatment characteristics for the investigated proton (MDACC) and photon (UKD, RUMC) cohorts.

		**UKD**	**MDACC**	**RUMC**
Gender				
	Female	51 (23%)	40 (47%)	74 (37%)
	Male	167 (77%)	46 (53%)	124 (63%)

Median age (range; years)		64 (33–79)	67 (39–80)	63 (36–83)
Smoker				
	No	72 (33%)	5 (6%)	0 (0%)
	Yes	146 (67%)	81 (94%)	198 (100%)

Tumor histology				
	Squamous Cell Carcinoma	116 (53%)	26 (30%)	78 (40%)
	Adenocarcinoma	85 (39%)	45 (52%)	83 (43%)
	Other	17 (8%)	15 (18%)	34 (17%)
Tumor location				
	Right upper lobe	71 (33%)	30 (35%)	80 (42%)
	Right middle lobe	9 (4%)	3 (4%)	8 (4%)
	Right lower lobe	26 (12%)	12 (14%)	18 (9%)
	Left upper lobe	56 (26%)	20 (23%)	45 (24%)
	Left lower lobe	12 (5%)	13 (15%)	16 (8%)
	Unknown	44 (20%)	8 (9%)	25 (13%)
Average GTV dose (STD)		66.9 Gy (±1.7 Gy)	70.3 Gy (±5.4 Gy)	65.9 Gy (±0.9 Gy)
Mean lung dose (STD)		15.7 Gy (±4.1 Gy)	16.5 Gy (±3.9 Gy)	16.1 Gy (±2.9 Gy)
Average V_20 Gy_ lungs (STD)		25.5 % (±6.7 %)	28.3 % (±6.9 %)	26.5 % (±5.4 %)
Average V_5 Gy_ lungs (STD)		55.8 % (±12.9 %)	39.0 % (±10.3 %)	61.0 % (±12.8 %)
Average heart dose (STD)		13.1 Gy (±9.9 Gy)	8.0 Gy (±7.4 Gy)	13.6 Gy (±8.8 Gy)
Average V_20 Gy_ heart (STD)		21.8 % (±19.2 %)	18.9 % (±14.7 %)	24.7 % (±18.8 %)
Average V_5 Gy_ heart (STD)		44.3 % (±29.3 %)	25.2 % (±17.2 %)	47.0 % (±29.3 %)
Average V_30 Gy_ heart (STD)		16.0 % (±15.8 %)	20.5 % (±10.9 %)	17.1 % (±14.2 %)
Average V_40 Gy_ heart (STD)		12.9 % (±13.9 %)	12.6 % (±11.2 %)	11.5 % (±10.3 %)
Average RV dose (STD)		10.0 Gy (±9.8 Gy)	2.7 Gy (±4.9 Gy)	8.1 Gy (±7.7 Gy)
Average RV V_5 Gy_ (STD)		39.0 % (±32.4 %)	9.5 % (±16.6 %)	36.2 % (±32.3 %)
Average LV dose (STD)		7.6 Gy (±9.0 Gy)	4.4 Gy (±9.1 Gy)	7.1 Gy (±8.3 Gy)
Average LV V_5 Gy_ (STD)		31.8 % (±31.7 %)	11.8 % (±20.4 %)	32.1 % (±34.5 %)
Average RA dose (STD)		15.3 Gy (±15.9 Gy)	11.5 Gy (±14.0 Gy)	13.4 Gy (±13.8 Gy)
Average RA V_5 Gy_ (STD)		47.8 % (±35.7 %)	30.8 % (±33.0 %)	46.3 % (±36.9 %)
Average LA dose (STD)		23.3 Gy (±15.8 Gy)	25.5 Gy (±19.0 Gy)	21.5 Gy (±14.4 Gy)
Average LA V_5 Gy_ (STD)		68.6 % (±30.8 %)	53.5 % (±31.9 %)	65.4 % (±34.2 %)
Cardiac Adverse Events (CTCAE v. 4.0)				
	Grade 0	n.a.	50 (58%)	108 (80%)
	Grade 1	n.a.	0 (0%)	14 (10%)
	Grade 2	n.a.	26 (30%)	5 (4%)
	Grade 3	n.a.	4 (5%)	6 (4%)
	Grade 4	n.a.	2 (2%)	2 (2%)
	Grade 5	n.a.	4 (5%)	0 (0%)
Radiation Pneumonitis				
	Grade 0	139 (64%)	59 (69%)	170 (86%)
	Grade 1	51 (23%)	6 (7%)	1 (1%)
	Grade 2	21 (10%)	12 (14%)	2 (1%)
	Grade 3	7 (3%)	9 (10%)	22 (10%)
	Grade 4	0 (0%)	0 (0%)	2 (1%)
	Grade 5	0 (0%)	0 (0%)	1 (1%)

Abbreviations: GTV − gross tumor volume, STD − standard deviation, RV – right ventricle, LV – left ventricle, RA – right coronary artery, LA – left coronary artery, n.a. – not available, UKD – Universitätsklinikum Dresden, MDACC − MD Anderson Cancer Center, RUMC- Radboud University Medical Center Nijmegen.

**Fig. 1:**
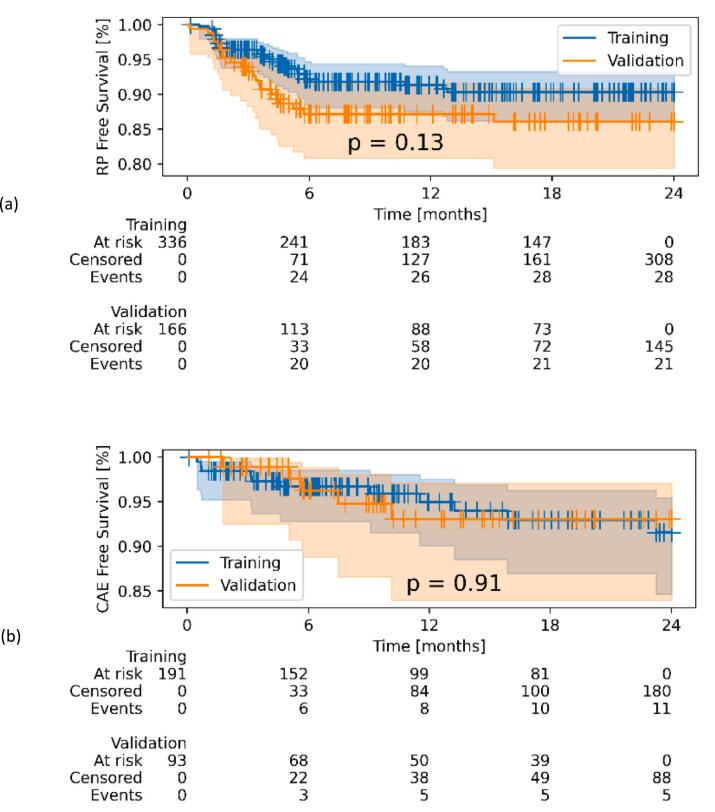
Kaplan Meier estimates with at-risk tables for the incidence of radiation pneumonitis events grade ≥ 2 (a) and cardiac adverse events grade ≥ 3 (b) in the training and the validation set. Log-rank tests between the different datasets yielded no significant differences (p-values given in the plot).

The mean dose to the entire heart was 13.1 Gy for patients from UKD, 8.0 Gy for patients from MDACC, and 13.6 Gy for patients from RUMC. Overall, the average doses to cardiac substructures were 23.4 Gy, 13.4 Gy, 6.4 Gy, and 6.9 Gy for LA, RA, LV, and RV respectively. Notably, mean RV doses of patients treated with protons (MDACC cohort) were significantly lower than for patients treated with photons (UKD and RUMC cohorts, p < 0.001). Further, significant differences between proton- and photon-cohorts were found for HeartV5Gy, LungV5Gy and LungV20Gy. Since data on the CAE for UKD was missing, only CAE data from MDACC and RUMC patients were assessed. The incidence of CAE grade ≥ 3 was 12 % and 6 % for MDACC and RUMC, respectively, and was not statistically significantly different between the training and validation set (Fig. 1).

Increased age did significantly correlate with RP grade ≥ 2 (p = 0.02) but not with CAE in patients. Patients with pulmonary comorbidities were also found to have higher risk of RP grade ≥ 2 (p = 0.01). History of smoking was significantly correlated with increased incidence of CAE grade ≥ 2 (p = 0.002). Tumor stage was significantly associated with CAE grade ≥ 3 (p = 0.02), however GTV volume did not significantly correlate with the endpoint.

**Fig. 2:**
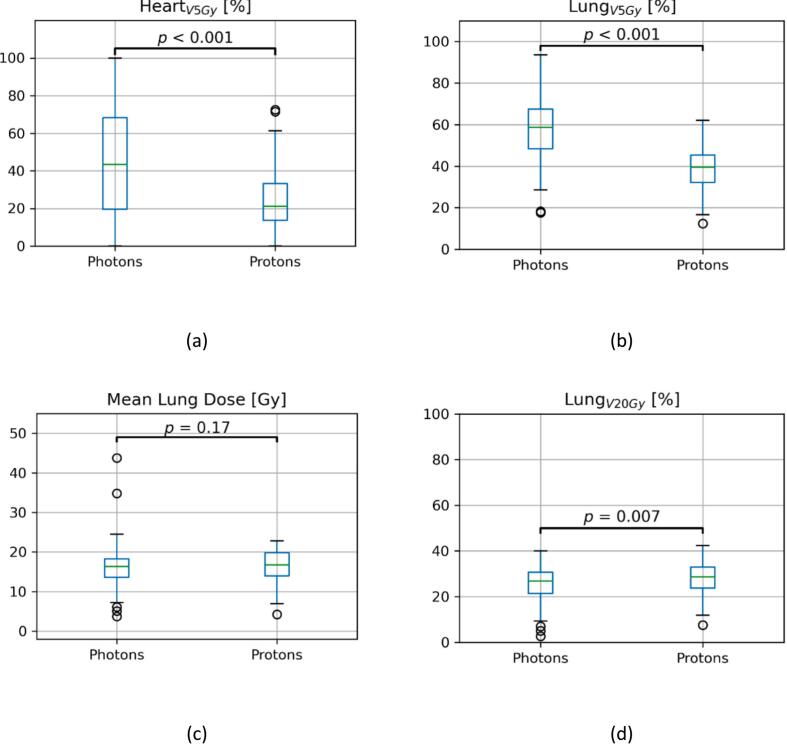
Boxplots of the parameters: (a) HeartV5Gy, (b) LungV5Gy, (c) MLD, and (d) LungV20Gy stratified for patients treated with photons (UKD and RUMC cohort) or protons (MDACC cohort); p-Values for mean-differences between the groups are provided using a two-sample *t*-test.

Models for the primary endpoint, CAE grade ≥ 3, based on HeartV5Gy, RVV5Gy, LVV5Gy, or LAV5Gy performed well, however, only showed a statistical trend and no significance (p_HeartV5Gy_ = 0.07, p_RVV5Gy_ = 0.06, p_LVV5Gy_ = 0.06, p_LAV5Gy_ = 0.06). Conversely, in the training cohort, the models predictive of CAE grade ≥ 3 based on parameters of the lungs (MLD, LungV20Gy, LungV5Gy) showed good association with the endpoint (p_MLD_ = 0.02, p_LungV20Gy_ = 0.02, p_LungV5Gy_ = 0.01). In the validation cohort, MLD and LungV20Gy were confirmed as relevant predictors (Table 2), however, the range of values for LungV20Gy was found to differ significantly between the proton and photon cohort (Fig. 2). When investigating the datasets independently, LungV20Gy could significantly predict CAE grade ≥ 3 for patients treated with protons (p_LungV20Gy_ = 0.45) but not with photons (p_LungV20Gy_ = 0.6). MLD could predict CAE grade ≥ 3 significantly in the proton cohort (p_MLD_ = 0.03). In the photon cohort a statistical trend could be observed (p_MLD_ = 0.06). For MLD, no significant differences between the parameter ranges were found. In multivariate analysis cardiac comorbidities were used together with MLD to predict CAE grade ≥ 3 well in training (p_MLD_ = 0.03, p_Cardiac Comorbidities_ = 0.04), however, this could not be confirmed in validation.

**Table 2:** Confidences and AUC for the different univariable binomial logistic-regression models for the training-set (*p < 0.05, ^**^p < 0.01) against the endpoint of CAE grade ≥ 3 (CTCAE v. 4.0); Confidence Intervals (CI) are based on the AUC bootstraps.

			**Training**	**Validation**
**Model**	**Parameter**	**p-Value**	**AUC (95 % CI)**	**AUC (95 % CI)**
1	MLD [Gy]	0.02*	0.69 (0.42–0.82)	0.77 (0.58–0.91)
2	MLD [Gy]	0.03*	0.72 (0.59–0.86)	0.45 (0.38–0.90)
	Cardiac Comorbidities	0.04*		
3	LungV20Gy	0.02*	0.69 (0.46–0.84)	0.77 (0.59–0.91)
4	LungV5Gy	0.01*	0.71 (0.51–0.87)	0.52 (0.38–0.98)
5	MHD [Gy]	0.34	0.60 (0.47–0.77)	0.66 (0.45–0.92)
6	HeartV20Gy	0.22	0.62 (0.47–0.77)	0.65 (0.49–0.90)
7	HeartV5Gy	0.07	0.67 (0.46–0.82)	0.63 (0.48–0.91)
8	HeartV30Gy	0.52	0.57 (0.46–0.73)	0.66 (0.47–0.93)
9	HeartV40Gy	0.86	0.53 (0.45–0.70)	0.66 (0.47–0.87)
10	RV mean dose [Gy]	0.09	0.68 (0.37–0.84)	0.47 (0.38–0.98)
11	RVV5Gy	0.06	0.71 (0.37–0.84)	0.49 (0.43–0.77)
12	LV mean dose [Gy]	0.09	0.73 (0.56–0.86)	0.63 (0.41–0.98)
13	LVV5Gy	0.06	0.72 (0.57–0.83)	0.48 (0.39–0.87)
14	RA mean dose [Gy]	0.33	0.60 (0.45–0.77)	0.70 (0.47–0.99)
15	RAV5Gy	0.08	0.64 (0.48–0.77)	0.68 (0.48–0.96)
16	LA mean dose [Gy]	0.25	0.59 (0.44–0.75)	0.64 (0.46–0.99)
17	LAV5Gy	0.06	0.69 (0.53–0.81)	0.62 (0.43–0.88)
18	Tumor location	0.28	0.57 (0.51–0.73)	0.37 (0.30–0.43)

Abbreviations: AUC − area under the receiver operating characteristic curve, MLD – mean lung dose, MHD – mean heart dose, VxGy – volume of organ receiving at least xGy, RV – right ventricle, LV – left ventricle, RA – right coronary artery, LA – left coronary artery.

Interestingly, the incidence of grade ≥ 3 CAE (12 % for protons, 6 % for photons) was higher in the proton cohort, even though patients treated with protons received significantly lower doses to most of the cardiac subregions investigated here (Fig. 2). The location of the primary tumor within the lungs as a predictive parameter provided no additional insight for CAE grade ≥ 3. Against the endpoint of CAE grade ≥ 2 the tumor location model showed significant performance in training, however this could not be confirmed in validation.

When testing model performance for the secondary endpoint, CAE grade ≥ 2, multiple parameters were identified as good predictors (p_HeartV5Gy_ = 0.03, p_LungV5Gy_ < 0.001; Supplement A). However, it was found that the range of these parameters differed significantly between the investigated datasets, invalidating these results (Fig. 2). For RP grade ≥ 2, no statistically significant predictors could be identified (see Supplement B). Of note, the incidences of grade ≥ 2 CAE (36 % for protons, 13 % for photons) and grade ≥ 2 RP (24 % for protons, 13 % for photons) were also higher in the proton cohort.

When investigating the treatment modalities separately for the endpoint of RP grade ≥ 2, in the proton cohort, MLD was the only parameter to significantly predict the endpoint (p_MLD_ = 0.01). No parameter was found to predict CAE grade ≥ 2 for protons or photons separately.

Finally, in our study, no evidence indicative of a specific sub-region of the heart being more vulnerable to the applied dose of photon or proton irradiation was found.

## 4 Discussion

Here, the predictability of cardiac and pulmonary toxicity using dosimetric parameters of the lungs and heart was investigated in three cohorts of NSCLC patients treated at three well-known international centers with photon or proton beam irradiation. Our goal was to find parameters valid for both photons and protons to predict cardiac and pulmonary toxicity, focusing on sub-regions of the heart and associated DHV parameters.

Until recently, cardiac toxicity was considered relevant for survivors of breast cancer and Hodgkin’s lymphoma. This, however, changed with RTOG 0617 [31], in which severe cardiac side effects were found to limit dose escalation for NSCLC patients [5], [8], [9]. Since then many studies investigated cardiac toxicity in NSCLC patients. The 2022 European Society of Cardiology (ESC) guidelines on cardio-oncology [10] summarizes the formation and effects of cardiac toxicity in patients undergoing radiochemotherapy for various solid tumors, including NSCLC and esophageal cancer. Risk factors for late cardiac adverse events include average radiation dose to the heart, chemotherapeutic agents and their dosage (anthracycline, doxorubicin, platinum-based agents) as well as preexisting cardiac toxicity.

Even though, in the data presented here, cardiac parameters showed a statistical trend in the training cohort, they did not perform well in the validation cohort. In contrast, the mean lung dose predicted cardiac adverse events grade ≥ 3 consistently throughout the different modalities and datasets. Therefore, in our dataset, the actual dose to the heart and its substructures appeared to be less relevant for the prediction of cardiac toxicity compared to the general dosimetric burden to the lungs, represented by the mean lung dose.

In literature, multiple studies indicate various cardiac dosimetric parameters to be predictive of cardiac toxicity, such as mean heart dose [16], HeartV10Gy [18], HeartV35Gy [17], the heart base [32], or in a multivariate analysis, dosimetric parameters of the left coronary artery [7]. In the datasets investigated here, these parameters did not significantly predict cardiac adverse events, which was confirmed when considering the datasets individually. In preclinical studies, van Luijk *et al.* [19] found the impact of cardiac dose to strongly depend on the irradiated lung volume. Furthermore, Wiedemann *et al.* [33] found preclinical evidence of radiation-induced lung damage to reduce right ventricular function. This was supported by Ghobadi *et al.* [20], reporting on cardiac damage to be explained by radiation-induced lung damage. These preclinical findings support our result on mean lung dose.

Interestingly, Ning *et al*. [17] found left-sided tumor location to be an indicator of increased cardiac toxicity. Therefore, we tested this in our study, and confirmed this against the endpoint of cardiac adverse events grade ≥ 2 in the training set, albeit not in the validation.

In the 2022 ESC guideline [10], chemotherapeutic agents and their dosage were also identified as risk factors for cardiac adverse events. In the three datasets investigated here, all patients received chemotherapeutic regiments, similar to those reported in the guideline, that were deemed a low risk for the development cardiac adverse events. Additionally, the mean heart doses for these patients were considered to be a moderate risk for the development of late cardiac toxicities. Therefore, for these low- to intermediate risk patients, possibly, a different set of parameters (dosimetric as well as others) governs the development of cardiac toxicity.

Of note, in our study, the incidences of both grade ≥ 3 and grade ≥ 2 cardiac adverse events as well as grade ≥ 2 radiation pneumonitis were higher in the proton cohort compared to the photon cohort, despite the lower radiation doses to heart and lungs. This could be due to a more thorough documentation of treatment-associated toxicity by the treating physicians, since for RUMC, toxicity follow-up was primarily focused around the lungs and esophagus [34]. In the MDACC publication, Gjyshi and Liao [35] also mention the possibility of a learning curve for physicians, physicists, and nurses working with passive-scattered proton therapy. Furthermore, patients from MDACC could have had more cardiac comorbidities, and therefore an increased risk for developing cardiac adverse events as well as radiation pneumonitis. Unfortunately, cardiac comorbidity data was not available for the MDACC patients, such that this hypothesis could not be confirmed.

Our study holds several limitations: (1) the differences in contouring of subregions of the heart between clinicians (MDACC dataset) and machine learning algorithms (RUMC and UKD dataset); (2) inclusion of different cardiac events in the investigated endpoint, possibly mixing up multiple dose–response relationships in our results; (3) differing treatment modalities between institutes, i.e. photons versus protons in addition to the fact that for MDACC patients, the 3D dose distributions were not available but the DVH data instead.

In summary, none of the cardiac parameters predicted cardiac adverse events in this multi-centric cohort, but the mean lung dose did. Moreover, for reasons not understood, patients treated with passive-scattered proton therapy had increased toxicity, even though the actively scanned technique as well as improved toxicity reporting in the proton field may explain this finding.

**Ethics Committee of TU Dresden Vote Number: BO-EK-251062024**.

## CRediT authorship contribution statement

**Albrecht Weiß:** Conceptualization, Software, Formal analysis, Investigation, Data curation, Writing – original draft, Visualization. **Steffen Löck:** Methodology, Writing – review & editing. **Ting Xu:** Resources, Writing – review & editing. **Zhongxing Liao:** Resources, Writing – review & editing. **Miguel Garrett Fernandes:** Resources, Data curation, Writing – review & editing. **René Monshouwer:** Resources, Writing – review & editing. **Johan Bussink:** Resources, Writing – review & editing. **Esther G.C. Troost:** Conceptualization, Methodology, Writing – review & editing, Supervision, Project administration.

## Declaration of Competing Interest

The authors declare that they have no known competing financial interests or personal relationships that could have appeared to influence the work reported in this paper.
