# Supplementary material for: Prediction for cardiac and pulmonary toxicity in a multicentric cohort of advanced stage NSCLC patients using sub-regions of the heart
Source: Clin Transl Radiat Oncol. 2025 Apr 1;53:100952. doi: 10.1016/j.ctro.2025.100952 (PMC12004370; doi:10.1016/j.ctro.2025.100952)
Supplement: Supplementary Data 1 [file mmc1.docx]

## Supplement A

Confidences and AUC for the different univariable binomial logistic-regression models for the training-set (* p < 0.05, ** p < 0.01, *** p < 0.001) against the endpoint of CAE grade ≥ 2 (CTCAE v. 4.0); Confidence Intervals (CI) are based on the AUC bootstraps

|  |  |  | **Training** | **Validation** |
| --- | --- | --- | --- | --- |
| **Model** | **Parameter** | **p-Value** | **AUC (95% CI)** | **AUC (95% CI)** |
| 1 | MLD [Gy] | 0.94 | 0.51 (0.46-0.64) | 0.54 (0.46-0.69) |
| 2 | MLD [Gy] | 0.93 | 0.51 (0.44-0.66) | 0.60 (0.48-0.76) |
|  | Cardiac Comorbidities | 0.86 |  |  |
| 3 | LungV20Gy | 0.98 | 0.50 (0.46-0.64) | 0.55 (0.43-0.70) |
| 4 | LungV5Gy | <0.001*** | 0.74 (0.64-0.83) | 0.64 (0.43-0.82) |
| 5 | MHD [Gy] | 0.70 | 0.48 (0.44-0.64) | 0.68 (0.52-0.81) |
| 6 | HeartV20Gy | 0.62 | 0.49 (0.45-0.63) | 0.69 (0.50-0.82) |
| 7 | HeartV5Gy | 0.03* | 0.61 (0.42-0.72) | 0.70 (0.52-0.83) |
| 8 | HeartV30Gy | 0.94 | 0.52 (0.45-0.64) | 0.67 (0.46-0.81) |
| 9 | HeartV40Gy | 0.21 | 0.57 (0.48-0.69) | 0.66 (0.44-0.80) |
| 10 | RV mean dose [Gy] | 0.03* | 0.65 (0.41-0.76) | 0.70 (0.50-0.84) |
| 11 | RVV5Gy | 0.007** | 0.66 (0.44-0.76) | 0.65 (0.49-0.78) |
| 12 | LV mean dose [Gy] | 0.74 | 0.60 (0.45-0.73) | 0.73 (0.53-0.90) |
| 13 | LVV5Gy | 0.07 | 0.60 (0.47-0.70) | 0.67 (0.52-0.84) |
| 14 | RA mean dose [Gy] | 0.20 | 0.61 (0.44-0.73) | 0.67 (0.41-0.83) |
| 15 | RAV5Gy | 0.04* | 0.63 (0.49-0.74) | 0.66 (0.41-0.82) |
| 16 | LA mean dose [Gy] | 0.30 | 0.54 (0.46-0.67) | 0.56 (0.44-0.73) |
| 17 | LAV5Gy | 0.27 | 0.58 (0.47-0.69) | 0.61 (0.40-0.76) |
| 18 | Tumor location | 0.05* | 0.59 (0.51-0.69) | 0.52 (0.37-0.67) |

Abbreviations: AUC - area under the receiver operating characteristic curve, MLD – mean lung dose, MHD – mean heart dose, VxGy – volume of organ receiving at least xGy, RV – right ventricle, LV – left ventricle, RA – right coronary artery, LA – left coronary artery

## Supplement B

Confidences and AUC for the different univariable binomial logistic-regression models for the training-set (* p < 0.05, ** p < 0.01) against the endpoint of RP grade ≥ 2 (CTCAE v. 4.0); Confidence Intervals (CI) are based on the AUC bootstraps

|  |  |  | **Training** | **Validation** |
| --- | --- | --- | --- | --- |
| **Model** | **Parameter** | **p-Value** | **AUC (95% CI)** | **AUC (95% CI)** |
| 1 | MLD [Gy] | 0.43 | 0.55 (0.45-0.64) | 0.66 (0.48-0.78) |
| 2 | MLD [Gy] | 0.41 | 0.56 (0.48-0.66) | 0.62 (0.45-0.71) |
|  | Cardiac Comorbidities | 0.44 |  |  |
| 3 | LungV20Gy | 0.30 | 0.56 (0.46-0.66) | 0.63 (0.43-0.76) |
| 4 | LungV5Gy | 0.40 | 0.54 (0.48-0.63) | 0.65 (0.46-0.67) |
| 5 | MHD [Gy] | 0.83 | 0.50 (0.46-0.60) | 0.59 (0.45-0.73) |
| 6 | HeartV20Gy | 0.79 | 0.50 (0.47-0.60) | 0.59 (0.46-0.73) |
| 7 | HeartV5Gy | 0.51 | 0.48 (0.46-0.60) | 0.56 (0.46-0.69) |
| 8 | HeartV30Gy | 0.86 | 0.52 (0.47-0.61) | 0.58 (0.43-0.73) |
| 9 | HeartV40Gy | 0.68 | 0.52 (0.47-0.61) | 0.58 (0.43-0.72) |
| 10 | RV mean dose [Gy] | 0.48 | 0.54 (0.45-0.63) | 0.52 (0.45-0.66) |
| 11 | RVV5Gy | 0.37 | 0.54 (0.46-0.63) | 0.52 (0.44-0.65) |
| 12 | LV mean dose [Gy] | 0.63 | 0.54 (0.46-0.63) | 0.49 (0.42-0.66) |
| 13 | LVV5Gy | 0.37 | 0.53 (0.47-0.62) | 0.52 (0.43-0.67) |
| 14 | RA mean dose [Gy] | 0.88 | 0.50 (0.45-0.61) | 0.56 (0.46-0.71) |
| 15 | RAV5Gy | 0.89 | 0.49 (0.47-0.61) | 0.53 (0.45-0.69) |
| 16 | LA mean dose [Gy] | 0.86 | 0.51 (0.47-0.60) | 0.55 (0.46-0.70) |
| 17 | LAV5Gy | 0.99 | 0.49 (0.46-0.60) | 0.54 (0.47-0.69) |
| 18 | Tumor location | 0.06 | 0.57 (0.50-0.65) | 0.51 (0.41-0.61) |

Abbreviations: AUC - area under the receiver operating characteristic curve, MLD – mean lung dose, MHD – mean heart dose, VxGy – volume of organ receiving at least xGy, RV – right ventricle, LV – left ventricle, RA – right coronary artery, LA – left coronary artery
